# Transcriptome Analysis Provides Insights into Copulation, Fertilization, and Gestation in *Sebastes schlegelii*

**DOI:** 10.3390/genes13101812

**Published:** 2022-10-07

**Authors:** Xueying Wang, Ning Zhao, Tao Wang, Shuran Du, Qinghua Liu, Jun Li

**Affiliations:** 1 CAS and Shandong Province Key Laboratory of Experimental Marine Biology, Center for Ocean Mega-Science, Institute of Oceanology, Chinese Academy of Sciences, Qingdao 266071, China; 2 Laboratory for Marine Biology and Biotechnology, Qingdao National Laboratory for Marine Science and Technology, Qingdao 266071, China; 3 University of Chinese Academy of Sciences, Beijing 100049, China; 4 Qingdao Agricultural University, Qingdao 266237, China

**Keywords:** *S. schlegelii*, copulation, fertilization, embryogenesis, RNA-seq

## Abstract

Among the viviparous marine teleosts of China, the black rockfish (*Sebastes schlegelii* Hilgendorf) is one of the most economically important. In addition to copulation and internal fertilization, it features lengthy sperm storage in the female ovary as well as a high rate of abortion. A network of gene regulation is necessary for these processes. To elucidate the mechanisms of copulation, fertilization, and gestation, it is essential to determine the genetic basis of viviparous teleost oogenesis and embryogenesis. In this study, we analyzed the transcriptome of the ovary during different developmental phases to investigate the dynamic changes that occur. We constructed 24 ovary transcriptomes. In order to investigate the regulation of embryogenesis, differentially expressed genes (DEGs) with specific expression patterns were subjected to gene ontology annotation, pathway analyses, and weighted gene co-expression network analysis (WGCNA). The up-regulated genes were significantly enriched in focal adhesion, regulation of the actin cytoskeleton, Wnt, and ECM-receptor interaction signaling pathways. As a result of our study, we provide omics evidence for copulation, fertilization, and gestation in viviparous marine teleosts. Decoding the *S. schlegelii* gene regulation network, as well as providing new insights into embryogenesis, is highly valuable to researchers in the marine teleost reproduction sciences.

## 1. Introduction

Oogenesis includes oogonia mitosis, proliferation, oocyte meiosis, growth, and maturation. In these processes, there are plentiful nutritive ingredients, RNA and protein accumulation, and maternal preparation for fertilization [1]. Teleosts have diverse reproductive strategies. For oviparous teleosts, egg maturation, ovulation, and fertilization occur externally; in contrast, for viviparous teleosts, sperm is transferred into females by specific copulatory organs and then stored, activated, and fertilized internally [2]. 

In recent years, RNA-seq techniques have attracted attention to reveal the molecular basis of oogenesis in fish, including Japanese flounder (*Paralichthys olivaceus*) [3], ricefield eel (*Monopterus albus*) [4], orange-spotted grouper (*Epinephelus coioides*) [5], Chinese sturgeon (*Acipenser sinensis*) [6], and so on. Such studies have revealed genes and pathways involved in steroid regulation, vitellogenesis, lipid droplet synthesis, oocyte development, and maturation [3,4,5,6]. More research on embryogenesis during gestation in females has been carried out in mammals and other viviparous species [7,8,9]. However, research on viviparous teleosts is limited [10]. With the rapid development of omic technology, there is an urgent need to increase the number of species for comparison in the gestation process through animals. 

*S. schlegelii* Hilgendorf is a viviparous marine teleost found in Japan, Korea, and China. The female reproductive cycle includes the ovary initiation, development, maturation, fertilization, and gestation stages. Copulation, fertilization, and gestation are the specific reproductive events in black rockfish for viviparity [11,12,13,14]. Previous studies have focused on the reproductive physiology regulation and structure basic for gestation. Gonadal development before fertilization was compared and provided insights into germ cell renewal and maturation [10]. Folliculogenesis and follicular placentation were reported as distinct structure preparation for gestation [15]. Multiple fetal nutritional patterns before parturition were also observed [16]. 

Our investigation in the present study focused on gonadal development throughout the entire breeding cycle of *S. schlegelii* using morphological and omics approaches to elucidate the molecular mechanisms involved in oogenesis and embryogenesis in viviparous teleosts. Here, we describe the different developmental stage features, identify a series of crucial genes and pathways, and provide details on the dynamic developmental characteristics of *S. schlegelii* copulation, fertilization, and gestation. This study provides valuable information on the reproductive biology of teleosts.

## 2. Materials and Methods

### 2.1. Sample

All animal experiments protocol was approved by the ethical committee of the Institute of Oceanology, Chinese Academy of Sciences.

Female *S. schlegelii* was obtained from the Nanshan market in Qingdao. The fish were obtained every half a month. The fish were anesthetized using MS-222 (100 μg/mL) before being weighed, measured, and photographed. The gonads were collected for histological analysis. According to the sampling time and gonad anatomical and histological characteristics, the ovaries were divided into different stages. The stages are II (initiation), III (development), IIIIV (development), V_1 (maturation), V_2 (fertilization), bla (blastula stage), gas (gastrula stage), lab (labor stage), and VI (regressed stage). Three biological replicates per stage were used for molecular analyses. 

### 2.2. Histological Analysis

The ovaries used in the histological analysis were fixed in Bouin’s solution for 24 h. They were then dehydrated with ethanol and clarified with xylene prior to being embedded in paraffin, followed by sectioning to a 5–7 μm thickness. The sections were stained with Hematoxylin Eosion (HE). Images were captured using a Nikon E80 microscope with a DS-Ri2 Imaging System (Nikon, Tokyo, Japan).

### 2.3. cDNA Library Construction and Transcriptome Analysis

Total RNA from the ovaries was obtained, and the quality was evaluated. In order to determine the extent of degradation and contamination of RNA, an agarose gel electrophoresis was performed. The purity of RNA (OD_260/280_ ratio) was tested using a Nanodrop Spectrophotometer (Thermo Fisher Scientific, Waltham, MA, USA). RNA concentrations were precisely quantified with a Qubit Fluorometer (Thermo Fisher Scientific), and the RNA integrity was accurately detected using the Agilent 2100 Bioanalyzer (Agilent, Santa Clara, CA, USA). Construction of the cDNA library, quality evaluation, and HiSeq sequencing was then performed.

The raw data were filtered to obtain clean reads. Based on the following criteria, reads with adapters, reads with greater than 10% N (N represents undetermined base information), and low-quality reads (reads in which qPHRED ≤ 20 bases account for more than 50% of the total read length) were removed. The expected number of fragments per kilobase of transcript sequence per million base pairs sequenced was used to calculate gene expression levels. Using the Illumina HiSeq platform (Illumina, San Diego, CA, USA), twenty-four cDNA libraries were constructed and sequenced. 

### 2.4. RT-qPCR Validation

Six genes (*cyp11a1*, *star*, *lhcgr*, *hsd17b3*, *itr*, and *cxcl12*) were chosen for validation of the DEG data using quantitative real-time PCR (RT-qPCR). The experiments used SYBR Green (Accurate Biotechnology Co. Ltd., Changsha, Hunan, China) detection in a CFX Connect Real-Time System (Bio-Rad, Hercules, CA, USA). The 18S rRNA gene was used as the reference gene in these experiments. An analysis of relative gene expression data was conducted using the 2^−ΔΔCt^ method. All reactions were performed in triplicate.

### 2.5. WGCNA

An expression network was constructed based on gene co-expression using the weighted gene co-expression network analysis (WGCNA) method, which was implemented using the WGCNA package in R version 4.2.0 [17] and the Novomagic, a free online platform for data analysis (https://magic.novogene.com).

### 2.6. Statistical Analysis

A statistical analysis was conducted using the SPSS 19.0 software package for Windows (SPSS Inc., Chicago, IL, USA). Data were expressed as the mean ± SD. Statistical significance was set at *p* < 0.05.

## 3. Results

### 3.1. Stages of Ovarian Development

**Figure 1:**
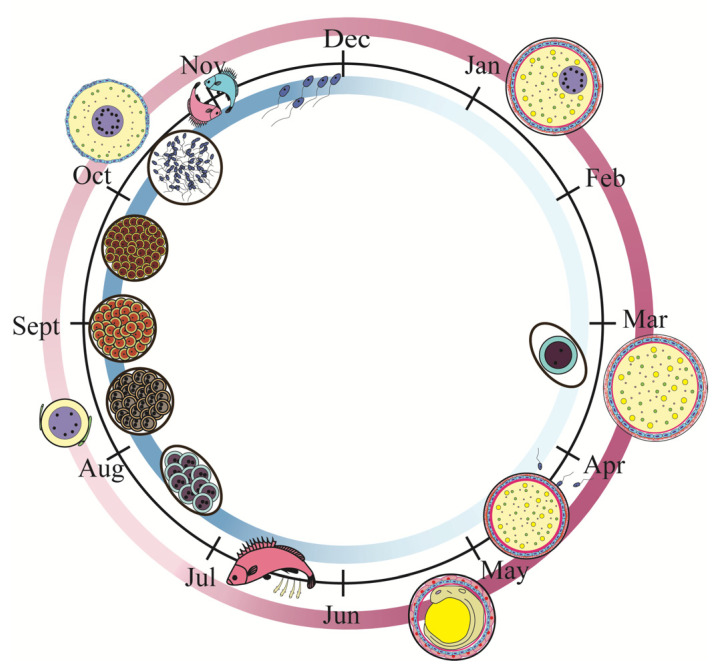
The pattern of the adult *S. schlegelii* Reproductive cycle. The male of *S. schlegelii* was mature and copulated from the middle of October to December. The female of *S. schlegelii* was maturation at about April. The fertilization in vivo occurred between April and May. The time was closely related to the water temperature. Aug–Oct: spermatogenesis; Oct–Dec: sperm maturation; Nov: copulation; Oct–Mar: vitellogenesis; Apr: fertilization; May–Jun: gestation. Mar: March; Apr: April; May: May; Jun: June; Aug: August; Oct: October; Nov: November; Dec: December.

**Figure 2:**
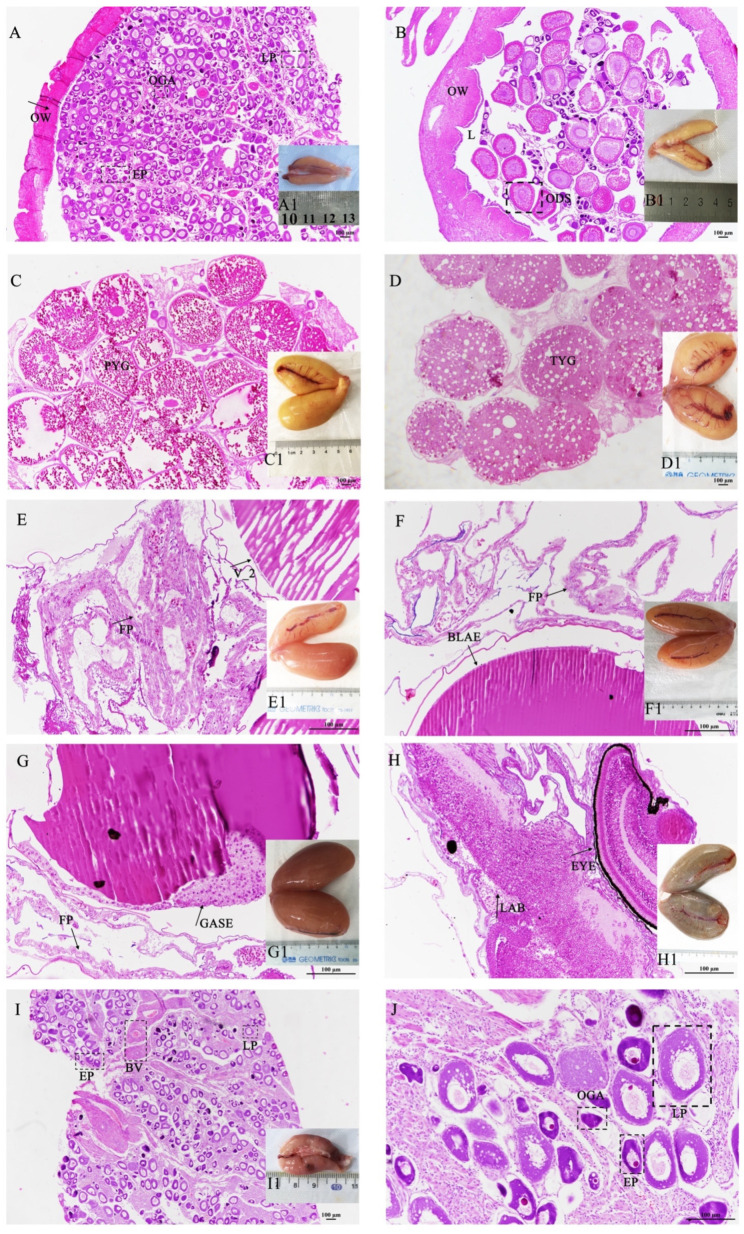
The characteristics of the ovary during the different developmental stages. (**A**) II stage, (**B**) III stage, (**C**) IIIIV stage, (**D**) V_1 stage, (E) V_2 stage, (**F**) bla stage, (**G**) gas stage, (**H**) lab stage, (**I**) VI stage, (**J**) high resolution of I image (scalebar, 100 μm). At the MII stage, the ovary shape was cylindrical, and its color was pink (**A1**). The cell type was mainly the oogonium and early peri-nucleolus (**A**). At the MIII stage, the ovary volume expanded, and the color was light yellow (**B1**). The cell types were mainly late peri-nucleolus oocytes (**B**). At the IIIIV stage, the yolk granules of oocytes constantly accumulated (**C**), and the ovary volume was expanded (**C1**). At the V_1 stage, the ovary matured and was rich with blood vessels (**D1**). The oocyte was filled with yolk granules, and lipid droplet vesicles were scattered (**D**). At the V_2 stage, the embryo was in the cleavage stage after fertilization (**E**,**E1**). At the bla stage, the embryo was in the blastula stage (**F**,**F1**). At the gas stage, embryos are in the gastrulation stage (**G**,**G1**). At the lab stage, the embryo is in the labor stage (**H**,**H1**). At this stage, the embryos have completed the hatching process and are born. The eyes were particularly obvious, and the ovaries appeared black. At the VI stage, the ovary shrank (**I1**), the cell type was mainly oogonia and early peri-nucleolus stage, and there was much more connective tissue, similar to that in oviparous teleosts after ovulation (**I**). J is the high resolution of I image (scalebar, 100 μm). OW: ovary wall; OGA, oogonia; EP, early peri-nucleolus stage oocyte; LP, late peri-nucleolus stage oocyte; ODS, oocytes at oil droplet stage; PYG, primary yolk globule stage; TYG, oocytes at tertiary yolk globule stage; FP, follicular placenta; BV, blood vessel; BLAE, bla stage embryo; GASE, gas stage embryo.

We determined the stage of ovarian development according to the developmental time, gonad index, and anatomical and histological characteristics of the ovary. *S. schlegelii* exhibits typical seasonal reproductive characteristics (Figure 1). In September, the ovary –initiated the development, and its shape was cylindrical and its color was pink (Figure 2(A1)). The cell type was mainly the oogonium and early peri-nucleolus stage oocyte (Figure 2A). This phase was defined as Phase II. In October, the ovary development and volume expanded, and the color was light yellow (Figure 2(B1)). The cell types were mainly late peri-nucleolus oocytes (Figure 2B). This phase was defined as phase III. In November and December, copulation occurs, and sperm is transferred to females. From December to February, the yolk granules of oocytes constantly accumulated (Figure 2C), and the ovary volume was expanded (Figure 2(C1)). This phase was defined as stage IIIIV. In March, the ovary matured and was rich with blood vessels (Figure 2(D1)). The oocyte was filled with yolk granules, and lipid droplet vesicles were scattered (Figure 2D). The phase was defined as V_1. From April to May, this phase enters the gestation period. The phases were defined according to the stage of embryo development observed under a stereo microscope. The embryo was in the cleavage stage after fertilization and was defined as V_2 (Figure 2E,E1). Embryos in the blastula stage were defined as the bla stage (Figure 2F,F1). Embryos in the gastrulation stage were defined as the gas stage (Figure 2G,G1). The embryo in the labor stage was defined as the lab stage (Figure 2H,H1). At this stage, the embryos have completed the hatching process and are born. Organ development was completed. The eyes were particularly obvious, and the ovaries appeared black. After pregnancy, the ovary shrank (Figure 2(I1)), the cell type was mainly oogonia and early peri-nucleolus stage oocyte, and there was much more connective tissue, similar to that in oviparous teleosts after ovulation (Figure 2I). The phase was defined as stage VI.

### 3.2. Sequence and Assembly Analysis

In this study, 24 cDNA libraries (II, III, IIIIV, V_1, V_2, bla, gas, lab, and VI) were constructed using total RNA from different developmental stages ovaries during oogenesis and embryogenesis. Two or three biological replicates were used for each stage. Standard quality control analyses were conducted to ensure that the RNA-seq data met the criteria for transcriptome analysis. We generated approximately 179.06 Gb of bases after Illumina HiSeq sequencing. The error rate (%), Q20, Q30, and GC content of the unigenes were 0.02–0.03%, 97.35%, 92.92%, and 50.21%, respectively. 

### 3.3. Differentially Expressed Gene Analysis

**Figure 3:**
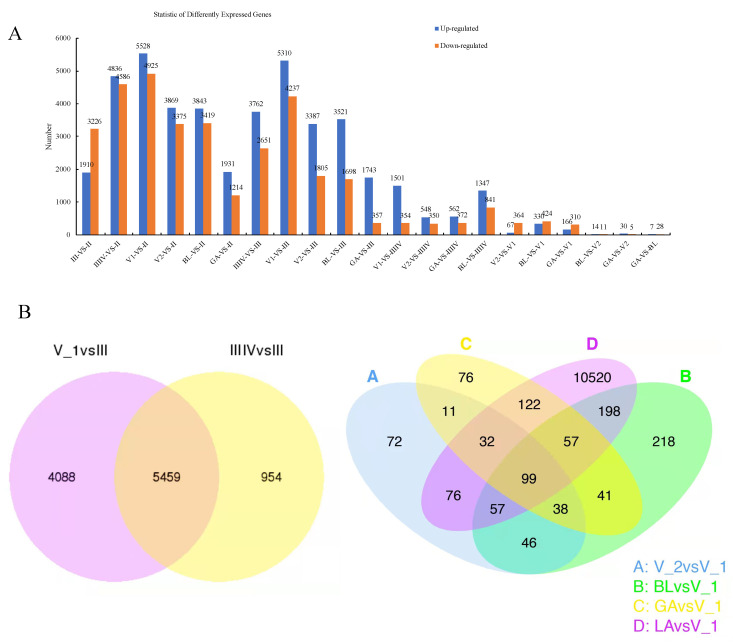
DEGs between the different ovarian developmental stages. (**A**) Summary of DEGs. The x-axis represents compared samples. The y-axis represents DEG numbers. The blue colour represents upregulated DEGs and the orange colour represents downregulated DEGs. (**B**) Venn diagram of ovarian transcripts from different reproductive phases.

The DEGs at different developmental stages are shown in Figure 3. The number of differentially expressed genes ranged from 7 to 5528 among the different developmental stages. The most differentially expressed genes were in V_1 vs. II or III. There were 4836 genes up-regulated and 4586 genes down-regulated between stages IIIIV and II, and 3762 genes were up-regulated, and 2651 genes were down-regulated between stages IIIIV and III. There were 67 up-regulated genes and 364 down-regulated genes between stages V2 and V1 (Figure 3A). 

There were 5459 common DEGs between stages V_1 and III, and IIIIV and III involved in copulation and ovarian maturation. Seventy-two DEGs were specifically between V_2 and V_1, and 218 DEGs were specifically between bla and V_1. Seventy-six DEGs were specifically between gas and V_1, and 10,520 DEGs were specifically between lab and V_1. There were 99 genes shared among the different comparison groups (Figure 3B).

**Figure 4:**
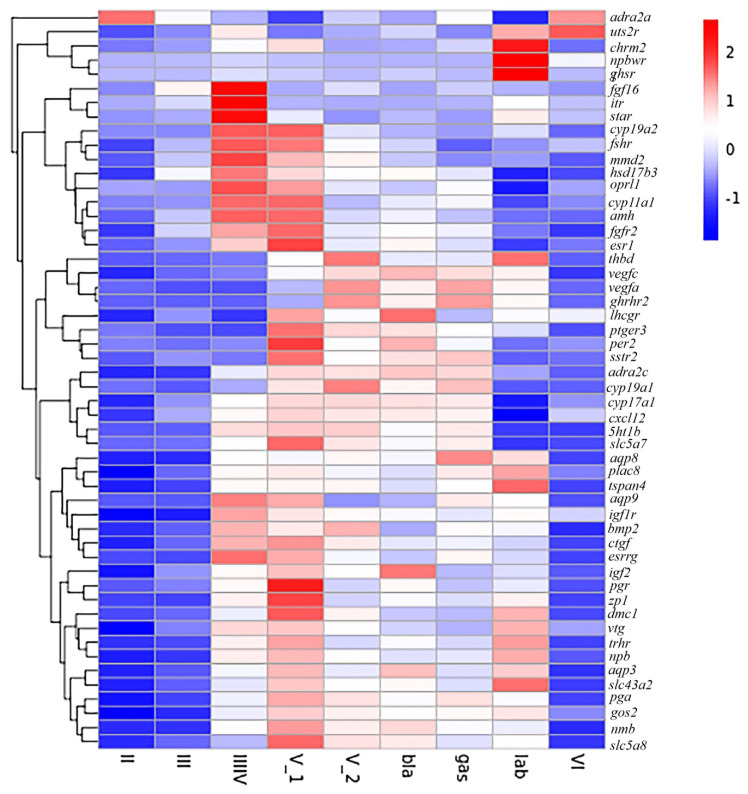
The heatmap of reproduction related DEGs expression pattern at different reproductive phases. The red and blue colors indicated up- and down-regulated transcripts, respectively.

The screened key DEGs related to reproduction between the developmental stages are listed in Table S1 (log2[fold] ≥ 4, *p* ≤ 0.05). During the oogenesis and maturation stages, the expression of steroid hormone metabolism and hormone receptor-related genes (*cyp19a1*, *star*, *cyp19a2*, *fshr*, *hsd17b3*, *cyp11a1*, *cyp17a1*, *esr1*, *lhcgr*, *ptger3*, *gnrhr2*, and *pgr*) was significantly up-regulated. The genes such as *adra2a*, *uts2r*, *chrm2*, *npbwr1*, *fgf16*, *thbd*, *5ht1b*, *itr*, *zp1*, *zp2*, *zp3*, *zp4*, *pga*, *hce1*, and *nmb*, may related to copulation, fertilization and gestation were presented in Figure 4 and Table S1.

### 3.4. Gene Ontology and Kyoto Encyclopedia of Genes and Genomes Enrichment Analysis of Differentially Expressed Genes

**Figure 5:**
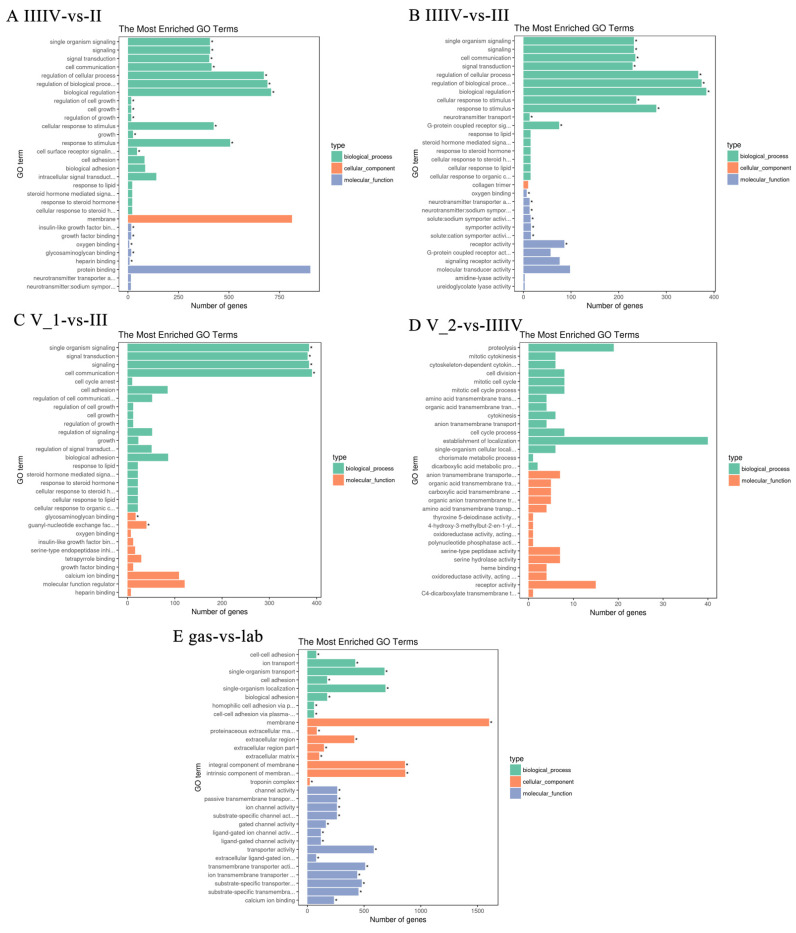
Gene ontology (GO) analysis of the DEG(s) with a two-fold difference. (**A**) IIIIV vs II, (**B**) IIIIV vs III, (**C**) V_1 vs III, (**D**) V_2 vs IIIIV, (**E**) gas vs lab. The x-axis shows the number of genes in each term. The y-axis shows the specific terms. The asterisk represents the corrected *p* value < 0.05 for each GO term.

We performed enrichment analyses using Gene Ontology (GO) and Kyoto Encyclopedia of Genes and Genomes (KEGG) to identify the biological function of DEGs in the copulation (IIIIV), ovarian maturation (V_1), fertilization (V_2), and gestation (bla, gas, and lab) developmental stages. The most enriched GO terms of IIIIV vs. II and IIIIV vs. III related to copulation in biological processes were regulation of cellular processes, biological regulation, and response to a stimulus. The most enriched GO terms of IIIIV vs. II and IIIIV vs. III related to copulation in molecular function were four types of receptor activity: receptors, G-protein coupled receptors, signaling receptors, and molecular transducers. The most enriched GO terms were signal transduction, cell communication, cell adhesion, calcium ion binding, and molecular function regulator. The most enriched GO terms for fertilization were proteolysis, cell cycle, the establishment of localization, and receptor activity. The most enriched GO terms for gestation were ion transport, membrane localization, and transmembrane transporters (Figure 5). 

**Figure 6:**
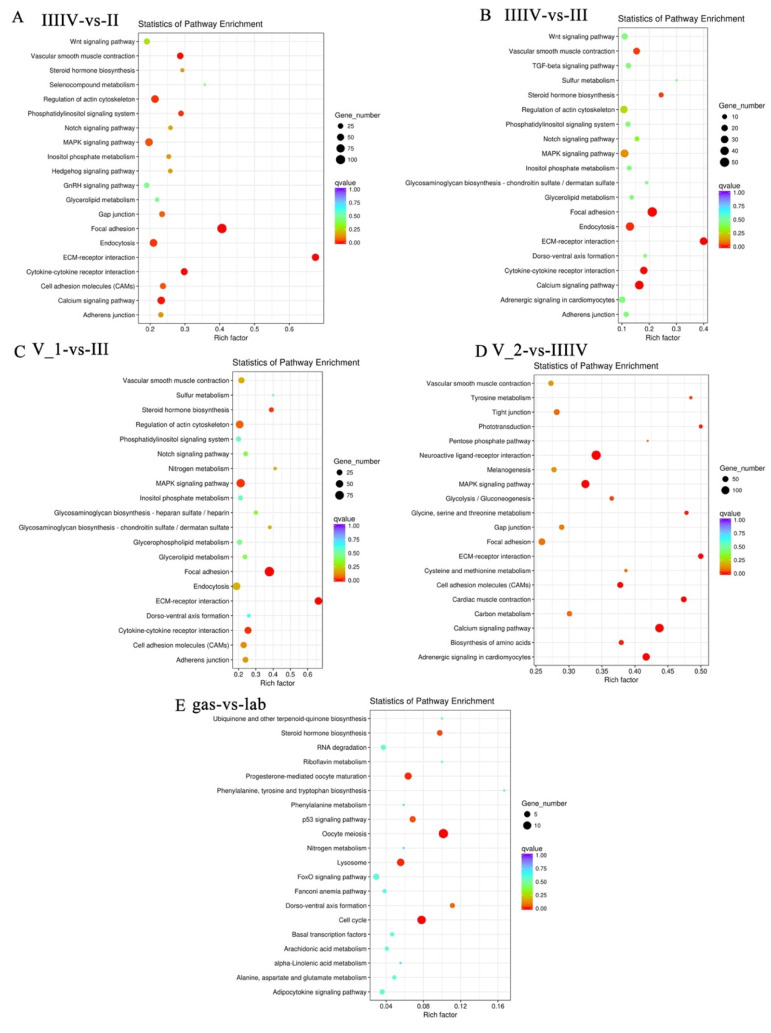
Scatter plots of enriched KEGG pathways for DEG(s) with a two-fold difference from the comparison of different stages. (**A**) IIIIV vs II, (**B**) IIIIV vs III, (**C**) V_1 vs III, (**D**) V_2 vs IIIIV, (**E**) gas vs lab. The rich factor is the ratio of the number of DEGs for a certain KEGG over the total genes in that pathway. *Q* value is the *p* value after correction for multiple testing. The color and size of the circles are *q* values and DEGs numbers, respectively.

The most enriched pathways during copulation and ovarian maturation were vascular smooth muscle contraction, focal adhesion, endocytosis, ECM-receptor interaction, signaling pathways involving calcium, cytokine-cytokine receptor interaction, regulation of the actin cytoskeleton, and MAPK pathways. The most enriched pathways during embryogenesis were the neuroactive ligand-receptor interaction, MAPK signaling, calcium signaling, adrenergic signaling in cardiomyocytes, steroid hormone biosynthesis, progesterone-mediated oocyte maturation, oocyte meiosis, lysosome, and cell cycle pathways (Figure 6). 

### 3.5. Validation of DEGs with RT-qPCR

**Figure 7:**
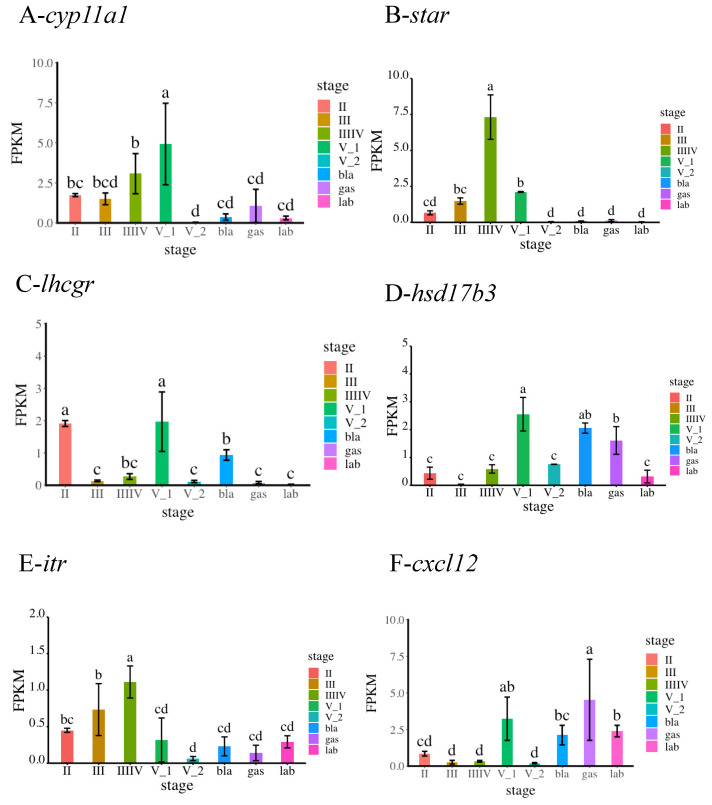
Validation of the DEGs by RT-qPCR. The expression levels of *cyp11a1*, *star*, *lhcgr*, *hsd17b3*, *itr, and cxcl12* during different developmental stages were detected by RT-qPCR. For reference genes, 18S were used for normalization of RT-qPCR data. Bars represent the standard deviation (SD). The x-axis indicates the developmental stage. The y-axis shows the relative expression level of genes. A: *cyp11a1*; B: *star*; C: *lhcgr*; D: *hsd17b3*; E: *itr*; F: *cxcl12*.

Gene expression levels for the selected genes (*cyp11a1*, *star*, *lhcgr*, *hsd17b3*, *itr*, and *cxcl12*) varied significantly (ANOVA, *p* < 0.05) depending on the reproductive stage. *cyp11a1* and star were significantly up-regulated from stage II to stage V_1, whereas *lhcgr* and *hsd17b3* were significantly up-regulated at stage V_1. The expression of *star* and *itr* was the highest at stage IIIIV during copulation. The expression of *cxcl12* was the highest at the gas stage during gestation (Figure 7). The RT-qPCR results showed a gene expression pattern similar to the RNA-seq analysis results during the different phases of reproduction.

### 3.6. Weighted Gene Co-Expression Network Analysis 

**Figure 8:**
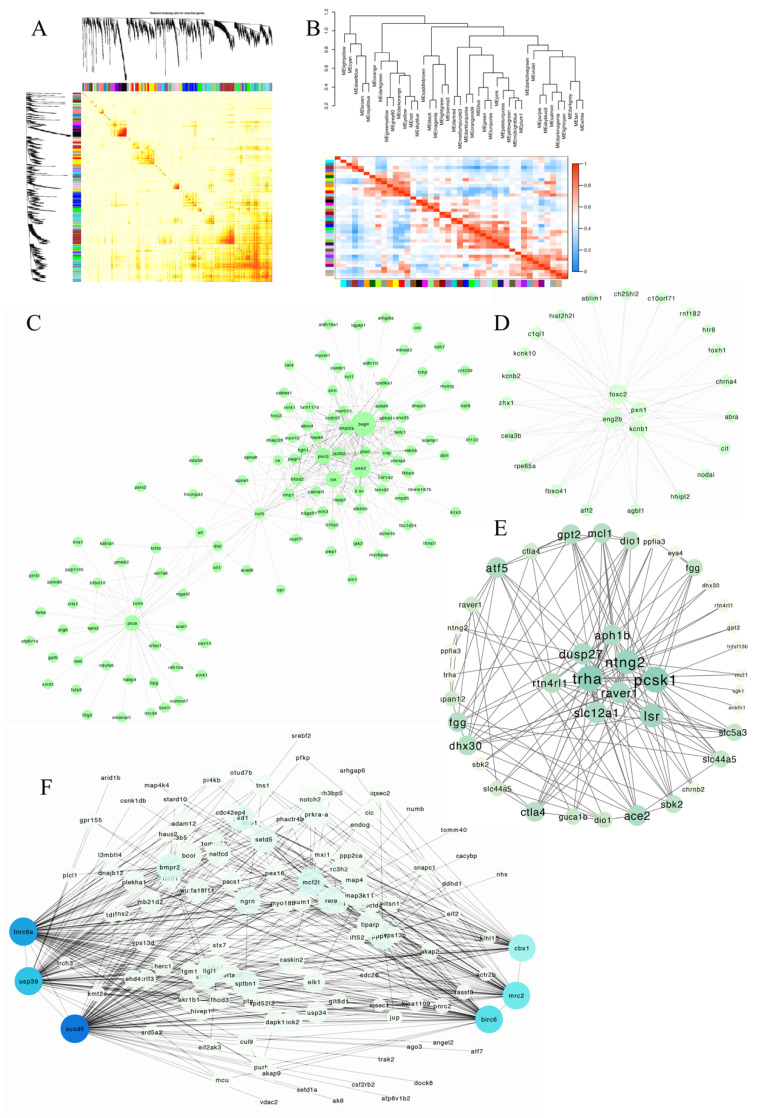
WGCNA analysis of RNA-seq and network visualization. (**A**): The heatmap of module-gene correlation. Each row and column represent a gene, and the darker the color of each point represents the stronger connectivity between the two genes. (**B**): The eigengene dendrogram of module membership. (**C**): Key genes related to copulation of the skyblue module. (**D**): The key genes related to fertilization of the blue module. (E): The key genes related to gestation of the green module. (**F**) The key genes related to the maturation and fertilization of purple modules. Note: The module was selected according to the relationship between the module and samples’ expression heatmap. (**C**–**F**) was a visualization by Cystoscope. The gene connectivity in each module was sorted by the connectivity value. The high connectivity genes were selected (the threshold value according to the gene numbers).

WGCNA is widely used in trait and gene correlation analyses. In this study, before WGCNA analysis, the selected gene set was screened and filtered to remove low-quality genes that had an unstable effect on the results, so that the network construction could be more accurate. Then, we constructed a hierarchical clustering tree, drew the power value curve, and divided the module. RNA-seq data were divided into modules (Figure 8A,B). According to the expression pattern, the genes *bag4*, *pex2*, *pkn3*, and *pcca*, which were involved in copulation, were significantly enriched in the sky-blue module (Figure 8C). of The genes, *foxc2*, *eng2b*, *pxn1*, *and kcnb1*, which were involved in gestation, were hub genes in the blue module (Figure 8D). Other groups genes *tnrc6a*, *usp39*, *susd6*, *cbx1*, *mrc2*, *birc6*, *trha*, *pcsk1*, and *lsr*, which were involved in fertilization, were significantly enriched in the green (Figure 8E) and purple modules (Figure 8F). 

## 4. Discussion

The marine teleost *S. schlegelii* is an economically important species. It has special reproductive characteristics, such as the asynchronization of male and female gonads development, copulation, and viviparity [18,19]. The urogenital papilla and follicular pseudoplacenta are specialized reproductive organs in this species [15]. The entire reproductive cycle of a female’s ovary includes oogenesis and gestational embryogenesis. Previous studies have either focused on oogenesis or parturition but did not cover the entire process. Oogenesis is the preparation of high-quality eggs for fertilization. Teleost viviparity has both oogenesis and gestational embryogenesis as reproductive characteristics. It is an ideal model to compare the entire process with other species. In this study, the gene expression pattern and molecular regulation network also reflected conservation during oogenesis and gestation among different species. This study used RNA-seq technology to reveal the genes and pathways involved in copulation, ovarian maturation, fertilization, and gestational embryogenesis. 

Hormones, including steroids and sex hormones, play important roles in regulating key processes during oogenesis and gestational embryogenesis. In this study, expression levels of *cyp19a1* and *cyp17a1* were both much higher at stages IIIIV, V_1, V_2, bla, and gas stages. *cyp11a1*, *cyp19a2*, *fshr*, *and lhcgr* showed the highest expression trends at stages IIIIV and V_1. Furthermore, changes in the steroid hormone levels of *S. schlegelii* ovaries have been reported in previous studies [15]. The hormone metabolic-related genes and hormone levels of E2 [15] both indicated that E2 continued to be synthesized during embryogenesis. It is well known that vitellogenin is synthesized by E2 during oogenesis in oviparous teleosts. Yolk accumulation is the main nutritional supply of eggs and embryos before hatching [20,21,22]. In this study, the embryo may receive nutrition from the mother’s body during pregnancy. Furthermore, the results of GO and KEGG enrichment analyses also showed that transport-, amino acid biosynthesis-, and carbon metabolism-related genes were significantly up-regulated during gestation.

Interesting, the sperm is an exogenous cue stimulus to the ovary. After copulation, the ovary quickly entered the growth and yolk accumulation stages. Copulation may influence oocyte development and maturation. It may be an adaptive strategy for female expenditure. During copulation, the focal adhesion, ECM-receptor interaction, calcium signaling, actin cytoskeleton regulation, and cytokine–cytokine receptor interaction pathways were significantly enriched. These pathways are involved in several cell developmental events during oogenesis, including cell proliferation, differentiation, growth, and migration. The process relies on cell interactions via cell focal adhesion. In focal adhesion, transmembrane proteins called integrins interact with extracellular ligands to form adhesive contacts between cells and the extracellular matrix. As well as anchoring cells to the substratum, focal adhesions also transmit biochemical signals [23,24,25,26]. The potential molecular gene network in oogenesis was conserved among different species with internal fertilization. Previous studies in Atlantic cod [27], yak [28], chickens [29], and insects [30] also reported similar pathways involved in oogenesis. In addition, in Drosophila [31], mating-induced oocyte maturation with calcium binding and transport proteins increased, as well as calcium signaling pathways that were also significantly enriched in the present study.

## Data Availability

The RNA-seq data generated during the current study were deposited in the NCBI Sequence Read Archive (SRA) database under BioProject number PRJNA867515.

## Supplementary Materials

The following are available online at https://www.mdpi.com/article/10.3390/genes13101812/s1, Table S1: Reproduction-related DEGs at different stages.
