# Supplementary material for: Transcriptome Analysis Provides Insights into Copulation, Fertilization, and Gestation in Sebastes schlegelii
Source: Genes (Basel). 2022 Oct 7;13(10):1812. doi: 10.3390/genes13101812 (PMC9601582; doi:10.3390/genes13101812)
Supplement: Supplementary file 1 [file genes-13-01812-s001.zip › genes-1900628-supplementary.pdf]

Supplementary-Table S1 Reproduction-related DEGs at different stages

Unigene name	Gene symbol	Padj	Log2 (fold)	Expression quantity	Trend
evm.TU.group18.1135	*adra2a*	0.017127	lnf	IIIIV-VS-II	up
evm.TU.group19.1125	*uts2r*	0.016681	lnf	IIIIV-VS-II	up
evm.TU.group22.516	*chrm2*	0.035585	lnf	IIIIV-VS-II	up
evm.TU.group4.285	*cyp19a1*	0.018537	lnf	IIIIV-VS-II	up
evm.TU.group5.88	*npbwr1*	0.047532	lnf	IIIIV-VS-II	up
evm.TU.group6.1217	*ghsr*	lnf	1.2298E-15	IIIIV-VS-II	up
evm.TU.group9.1070	*fgf16*	9.2794	2.537E-20	IIIIV-VS-II	up
evm.TU.group0.572	*itr*	7.08	6.8037E-06	IIIIV-VS-II	up
evm.TU.group19.455	*star*	6.9532	4.7244E-33	IIIIV-VS-II	up
evm.TU.group4.284	*cyp19a2*	6.5031	3.5787E-06	IIIIV-VS-II	up
evm.TU.group19.374	*fshr*	6.3578	3.5924E-39	IIIIV-VS-II	up
evm.TU.group16.385	*mmd2*	6.0584	1.1661E-09	IIIIV-VS-II	up
evm.TU.group7.357	*hsd17b3*	6.029	0.00001664	IIIIV-VS-II	up
evm.TU.group5.65	*oprl1*	5.6587	0.0025566	IIIIV-VS-II	up
evm.TU.group4.398	*cyp11a1*	5.4765	1.006E-21	IIIIV-VS-II	up
evm.TU.group3.816	*amh*	5.2263	8.4033E-34	IIIIV-VS-II	up
evm.TU.group12.748	*fgfr2*	5.169	5.5984E-37	IIIIV-VS-II	up
evm.TU.group17.556	esr1	4.8374	1.0098E-35	IIIIV-VS-II	up
evm.TU.group13.335	*thbd*	4.5722	2.2065E-11	IIIIV-VS-II	up
evm.TU.group23.295	*vegfc*	4.5107	9.7747E-09	IIIIV-VS-II	up
evm.TU.group9.1192	*vegfa*	4.3171	2.6437E-09	IIIIV-VS-II	up
evm.TU.group1.931	*gnrhr2*	4.2187	0.000031363	IIIIV-VS-II	up
evm.TU.group12.557	*lhcgr*	4.2146	2.9407E-17	IIIIV-VS-II	up
evm.TU.group3.487	*ptger3*	4.089	5.8082E-07	IIIIV-VS-II	up
evm.TU.group21.675	*per2*	4.066	1.6954E-33	IIIIV-VS-II	up
Novel01696	*sstr2*	lnf	0.0054441	IIIIV-VS-III	up
evm.TU.group18.1110	*adra2c*	lnf	0.049642	IIIIV-VS-III	up
evm.TU.group4.284	*cyp19a2*	7.2324	6.1346E-10	IIIIV-VS-III	up
evm.TU.group19.455	*star*	6.8959	4.9446E-45	IIIIV-VS-III	up
evm.TU.group1.343	*cyp19a1*	6.2384	1.1447E-61	IIIIV-VS-III	up
evm.TU.group0.572	*itr*	5.4764	2.5578E-06	IIIIV-VS-III	up
evm.TU.group12.557	*lhcgr*	5.0818	5.0522E-26	IIIIV-VS-III	up
evm.TU.group1.931	*gnrhr2*	4.5862	2.1034E-07	IIIIV-VS-III	up
evm.TU.group21.34	*cyp17a1*	4.4207	0.0010516	IIIIV-VS-III	up
evm.TU.group7.357	*hsd17b3*	4.1668	0.00001173	IIIIV-VS-III	up
evm.TU.group2.440	*cxcl12*	4.0685	7.883E-27	IIIIV-VS-III	up
evm.TU.group13.879	*5ht1b*	lnf	0.00032065	V_1-VS-II	up
evm.TU.group6.1217	*ghsr*	lnf	0.000023675	V_1-VS-II	up

evm.TU.group7.357	*hsd17b3*	7.9548	0.000037642	V_1-VS-II	up
evm.TU.group16.385	*mmd2*	7.473	1.9263E-17	V_1-VS-II	up
evm.TU.group9.1227_evm.TU.group9.1228	*slc5a7*	7.4473	3.8504E-17	V_1-VS-II	up
evm.TU.group13.940	*ghsr*	7.2135	0.009761	V_1-VS-II	up
evm.TU.group16.1286	*aqp8*	7.0565	2.6458E-06	V_1-VS-II	up
evm.TU.group19.455	*star*	7.0448	0.0059265	V_1-VS-II	up
evm.TU.group21.320	*plac8*	6.5904	0.000696	V_1-VS-II	up
evm.TU.group17.556	*esr1*	6.1698	4.9387E-77	V_1-VS-II	up
evm.TU.group4.398	*cyp11a1*	6.1618	1.3857E-13	V_1-VS-II	up
evm.TU.group12.537	*lhcgr*	5.4383	7.1732E-24	V_1-VS-II	up
evm.TU.group1.247	*tspan4*	5.3756	9.7397E-17	V_1-VS-II	up
evm.TU.group19.374	*fshr*	5.2995	4.6981E-09	V_1-VS-II	up
evm.TU.group1.456	*aqp9*	5.225	0.0006894	V_1-VS-II	up
evm.TU.group4.503	*igf1r*	5.201	0.000029739	V_1-VS-II	up
evm.TU.group6.797	*bmp2*	5.1953	3.1102E-12	V_1-VS-II	up
evm.TU.group5.1343	*ctgf*	5.0098	0.046568	V_1-VS-II	up
evm.TU.group21.34	*cyp17a1*	4.8139	6.3392E-17	V_1-VS-II	up
evm.TU.group17.761	*esrrg*	4.602	0.045916	V_1-VS-II	up
evm.TU.group4.68	*igf2*	4.5985	5.9372E-24	V_1-VS-II	up
evm.TU.group6.1235	*pgr*	4.5108	2.2961E-19	V_1-VS-II	up
evm.TU.group4.1088	*zp1*	4.0771	1.3931E-06	V_1-VS-II	up
evm.TU.group19.1023	*dmc1*	-4.9351	5.0971E-08	V_1-VS-II	down
evm.TU.group18.1135	*adra2a*	6.6301	0.0032896	V_1-VS-III	up
evm.TU.group18.1092	*adra2c*	lnf	0.038904	V_1-VS-III	up
evm.TU.group19.455	*star*	6.9679	0.00084422	V_1-VS-III	up
evm.TU.group3.490	*vtg*	6.3648	0.0052531	V_1-VS-III	up
evm.TU.group8.116	*trhr*	5.6285	1.6692E-17	V_1-VS-III	up
evm.TU.group16.1160	*npb*	5.0238	0.0055353	V_1-VS-III	up
evm.TU.group7.624	*aqp3*	8.8494	3.7937E-06	V_2-VS-II	up
evm.TU.group10.738	*slc43a2*	7.5253	4.3848E-33	V_2-VS-II	up
evm.TU.group21.320	*plac8*	5.4464	9.8576E-06	V_2-VS-II	up
evm.TU.group5.557	*pga*	5.3981	0.000037182	V_2-VS-II	up
evm.TU.group2.440	*cxcl12*	0.019531	4.6699	V_2-VS-II	up
evm.TU.group5.1229	*gos2*	0.022529	4.6682	V_2-VS-II	up
evm.TU.group3.490	*vtg*	0.028672	4.8528	V_2-VS-III	up
evm.TU.group4.460	*nmb*	0.00012312	lnf	V_2-VS-IIIIV	up
evm.TU.group0.1146	*slc5a8*	0.000030133	8.8554	V_2-VS-IIIIV	up

evm.TU.group4.1088	*zp1*	3.9446E-12	-7.1991	LA-VS-V_1	down
evm.TU.group5.557	*pga*	3.4722E-12	-6.5038	LA-VS-V_1	down
evm.TU.group11.261	*zp3*	8.1221E-15	-5.2062	LA-VS-V_1	down
evm.TU.group15.460	*zp4*	6.7778E-23	-4.5228	LA-VS-V_1	down
evm.TU.group17.229	*zp2*	2.5653E-22	-4.4441	LA-VS-V_1	down
evm.TU.group10.821	*zar1*	1.8638E-21	-4.398	LA-VS-V_1	down
Novel02618	*hce1*	1.4412E-08	-4.3445	LA-VS-V_1	down
